# Gangrene Secondary to Perineal Invasion by Rectal Cancer: A Case Report and Literature Review

**DOI:** 10.7759/cureus.76444

**Published:** 2024-12-26

**Authors:** Xinyu Li, Junqi Shan, Yanlai Sun

**Affiliations:** 1 School of Clinical Medicine, Shandong Second Medical University, Weifang, CHN; 2 Department of Surgical Oncology, Shandong Cancer Hospital and Institute, Shandong First Medical University, Shandong Academy of Medical Sciences, Jinan, CHN

**Keywords:** fistula, infection, invasion, perineum, rectal cancer

## Abstract

Colorectal cancer usually metastasizes through lymphatic, blood, and intraperitoneal implantation. However, rectal cancer combined with perineal invasion after treated with chemotherapy is very rare. The present case study is of a 53-year-old male patient with a history of rectal cancer who developed a recto-perineal fistula with redness, swelling, and pain in the scrotum after repeated chemotherapy. After a CT examination and investigative observation, the patient was diagnosed with a recto-perineal fistula. After surgical debridement and postoperative implant treatment, the patient's infection resolved, and the skin-grafting treatment worked well. The present case called attention to rectal cancer after being treated with chemotherapy.

## Introduction

Colorectal cancer is currently the third most common cancer in the world [1]. Rectal cancer usually metastasizes to the liver [2] and lungs [3] through lymph node metastasis or the blood pathway, abdominal implantation metastasis, and the remaining rare organs, such as the testes and ovaries [4]. After chemotherapy for rectal cancer, rectal cancer with perineal infiltration is rare and difficult to treat. Rectoperineal fistula formed by rectal cancer infiltrating the perineum leads to redness, swelling, pain, and other discomfort in the perineum, which seriously affects the patient’s quality of life. In this paper, a case of rectal perineal fistula after chemotherapy treatment for rectal cancer is reported to raise clinical attention to this disease and provide ideas for the clinical diagnosis and treatment of rectal perineal fistula in patients with rectal cancer.

## Case presentation

**Figure 1:**
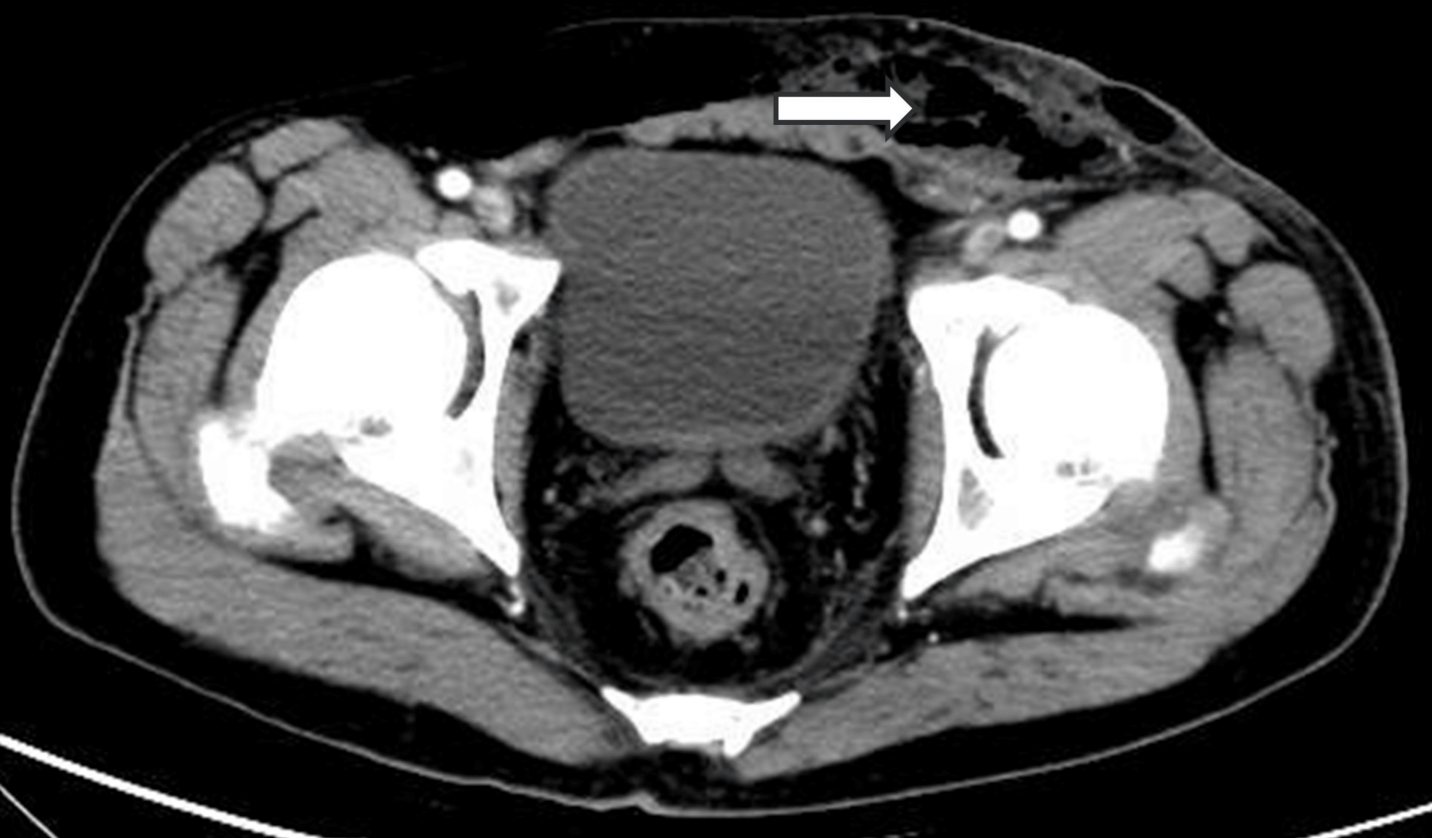
Pelvic CT of the left lower abdominal wall has infections and subcutaneous emphysema.

**Figure 2:**
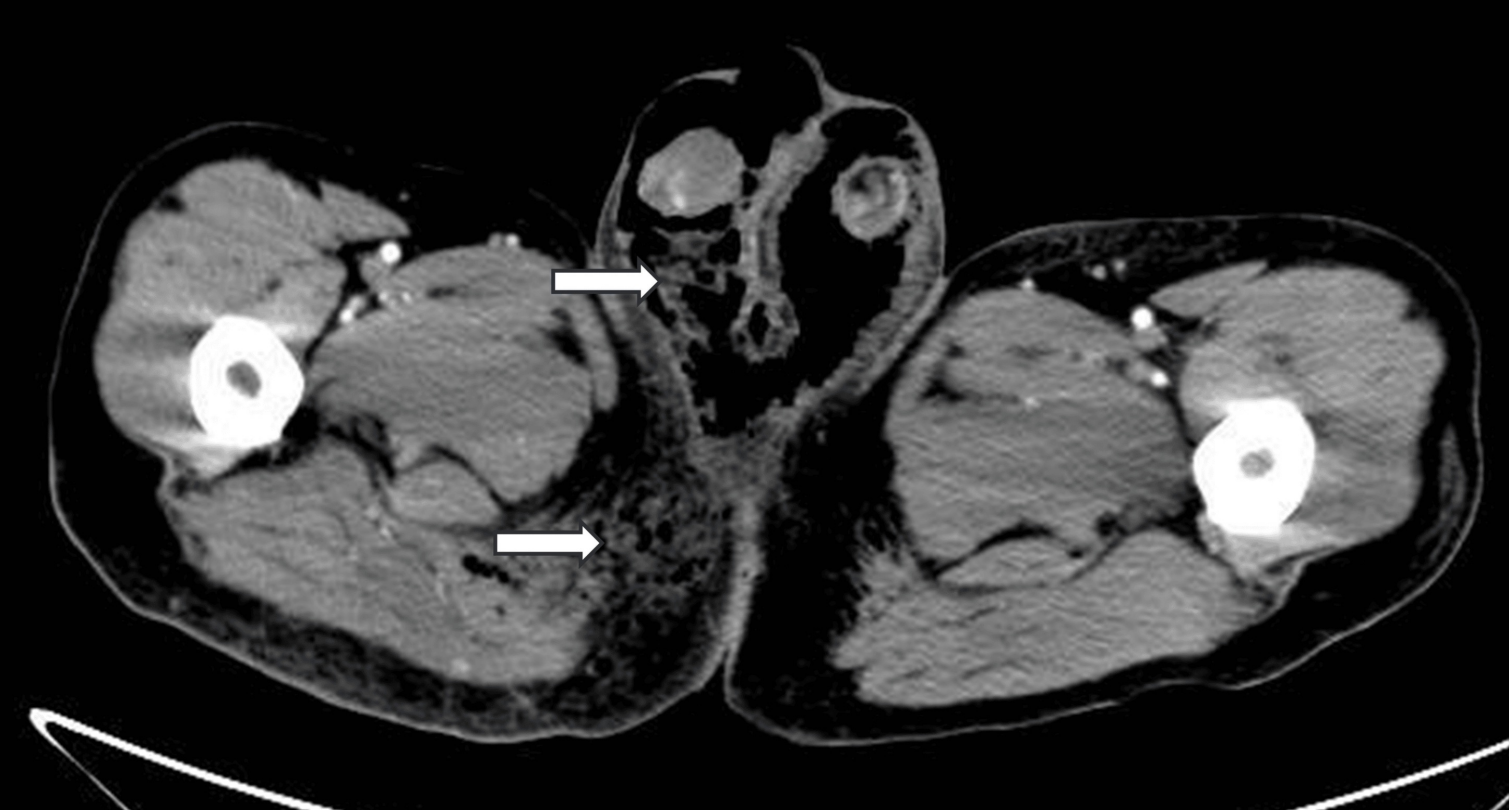
Pelvic CT of the left lower abdominal wall has right perineum infection.

**Figure 3:**
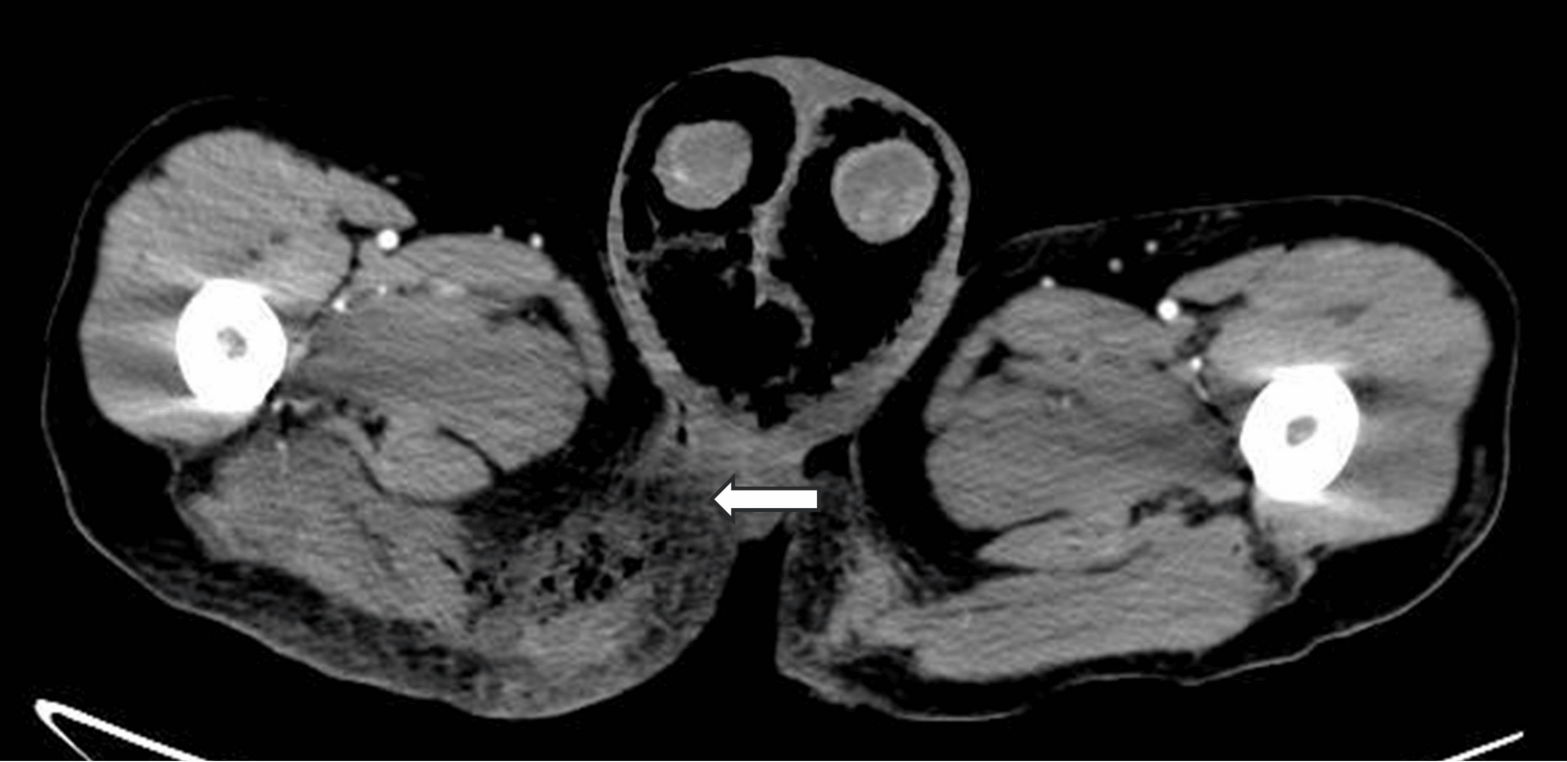
Pelvic CT of the left lower abdominal wall has buttock subcutaneous emphysema.

**Figure 4:**
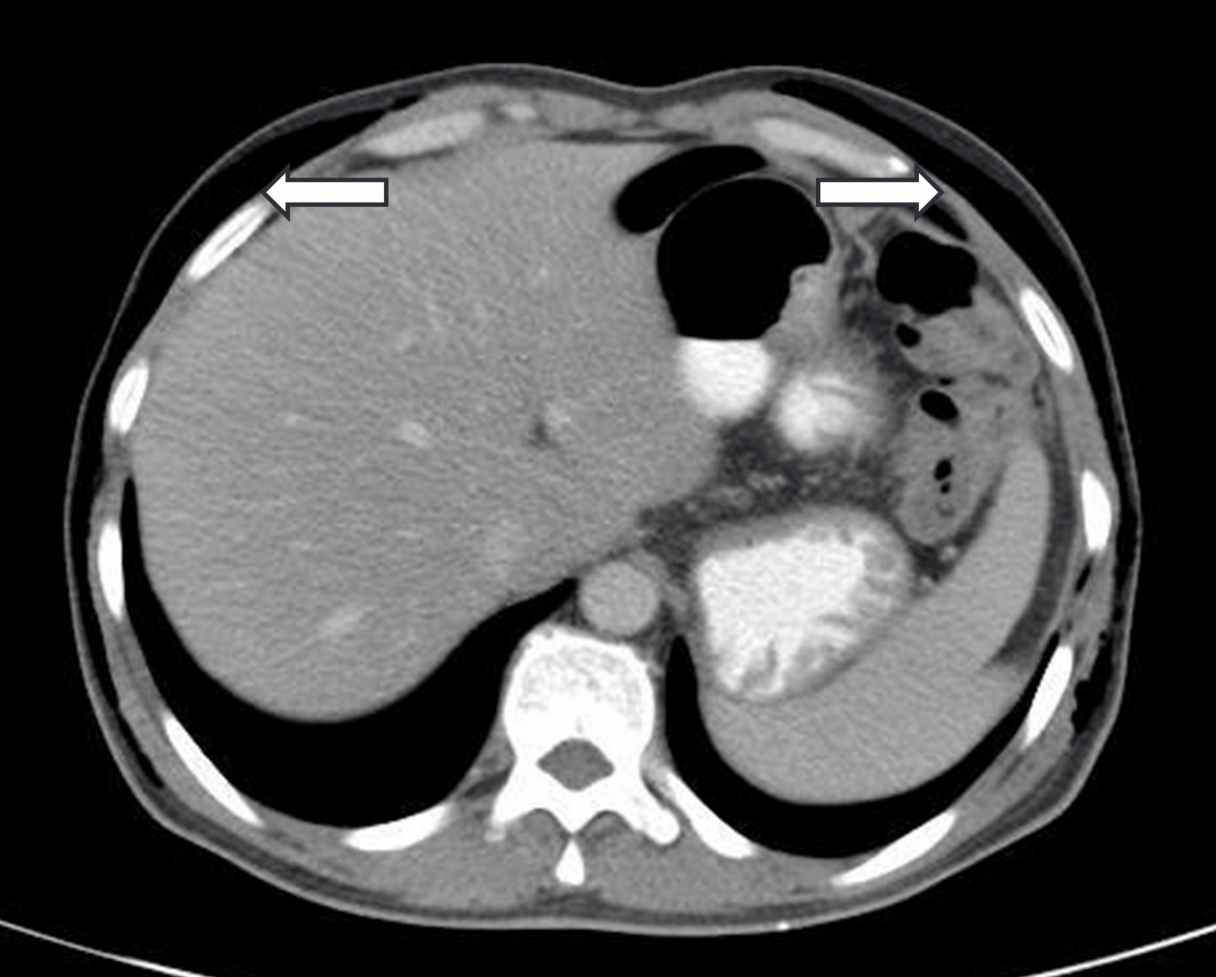
Subcutaneous emphysema of the chest wall.

**Figure 5:**
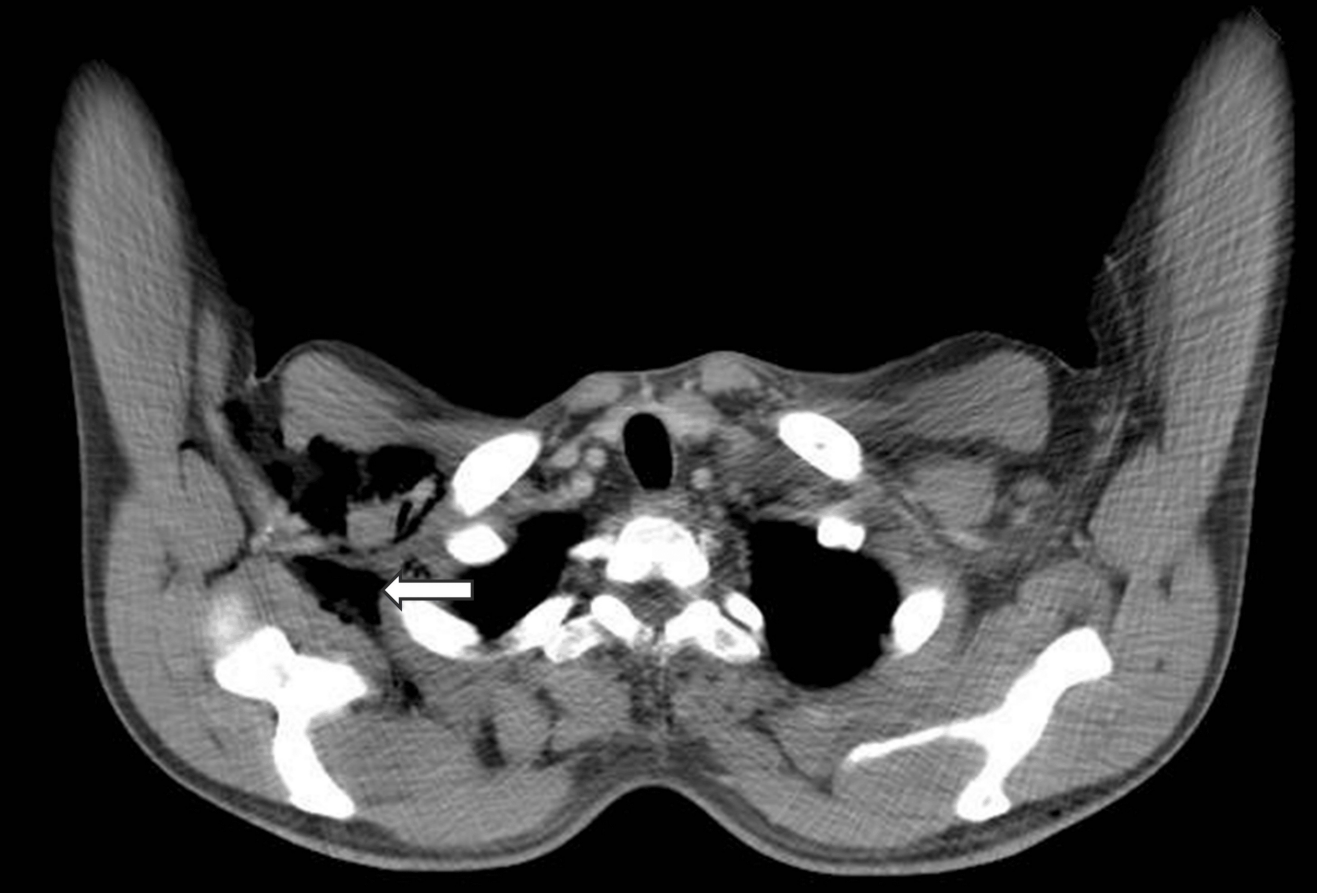
Subcutaneous emphysema of the abdominal wall.

A male patient, 53 years old, was admitted to the hospital on April 2023, with complaints of redness, swelling, and pain in the perineum and scrotum for five days after targeted combination chemotherapy for rectal cancer. The patient had a six-month history of rectal cancer (cT4bN+M1a, stage IVA, having three cycles of bevacizumab+ CapeOX regimen chemotherapy. Pelvic CT of the left lower abdominal wall showed infections and subcutaneous emphysema Figures 1-5).

**Figure 6:**
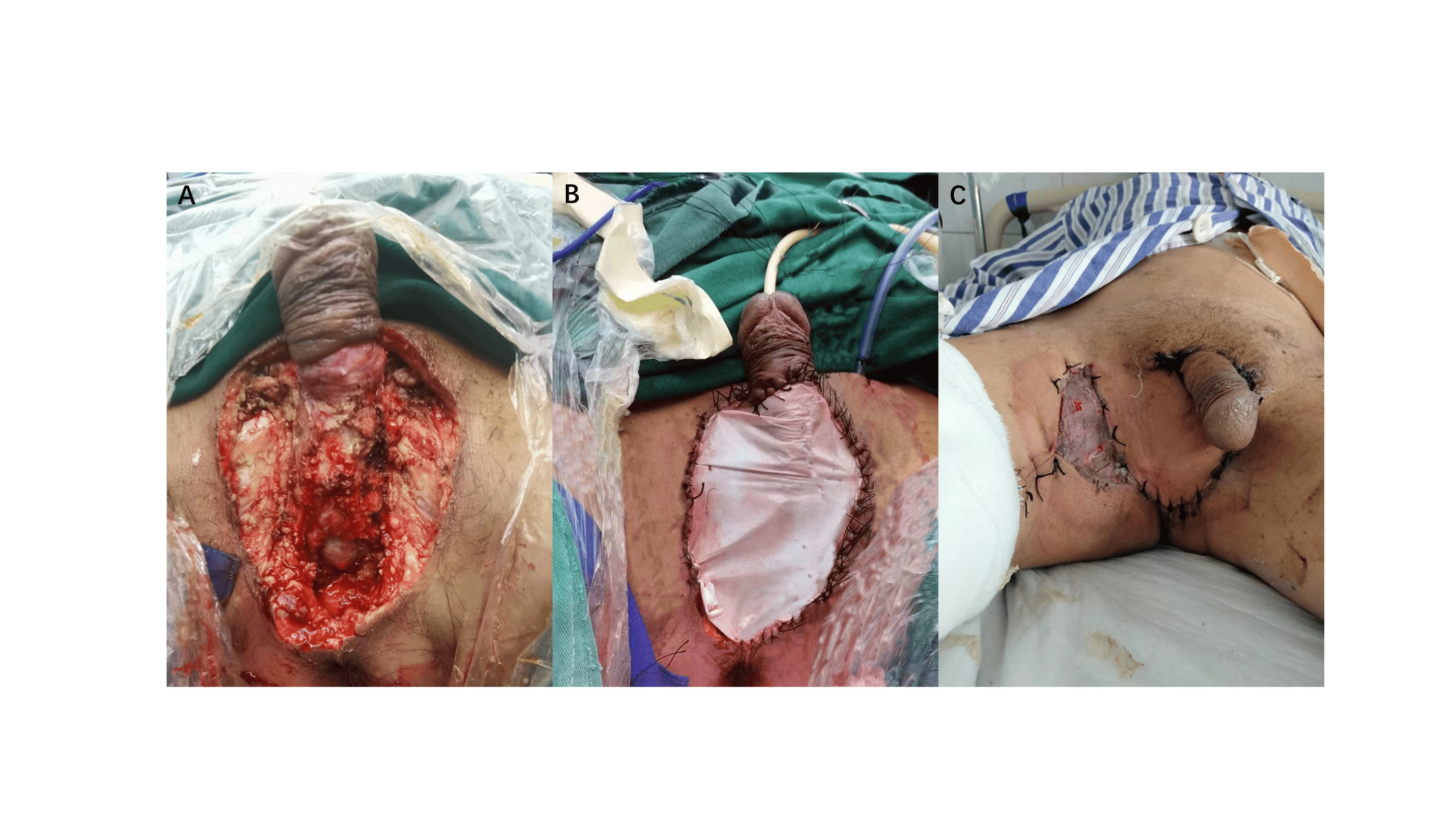
Bilateral orchiectomy perineum repair. A: Before biomaterials repair, B: After biomaterials repair, and C: Skin grafting status of the patient.

After completing the CT test, the patient was given a blood test. The procalcitonin test was 0.39 ng/L, the white blood cell count was 13.51 × 109/L, the neutrophil count was 11.77 × 109/L, the red blood cell count was 3.41 × 1012/L, the hemoglobin was 115 g/L, and the platelet count was 195 × 109/L. The procalcitonin test was 2.69 ng/L, and the B-type natriuretic peptide was 360.00 pg/m. Eventually, he underwent laparoscopic sigmoid colostomy, intestinal adhesion release, and bilateral orchiectomy perineum repair surgery (Figures 6A, 6B).

**Figure 7:**
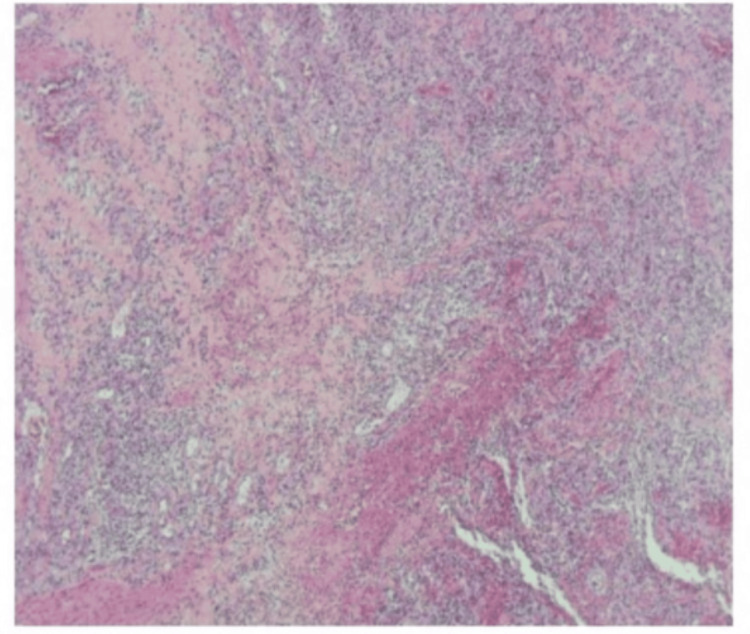
Pathological report. Bilateral testes and infected tissues of perineum: Fibrous tissue hyperplasia with massive acute and chronic inflammatory cell infiltration, vascular hyperplasia, congestion, focal necrosis, and small abscess formation.

**Figure 8:**
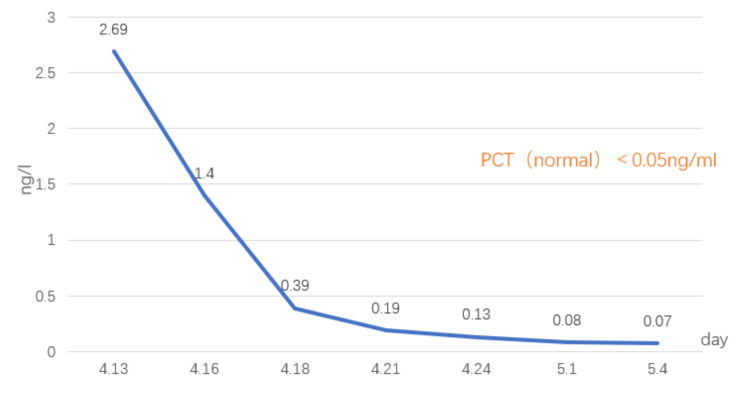
Procalcitonin monitoring.

**Figure 9:**
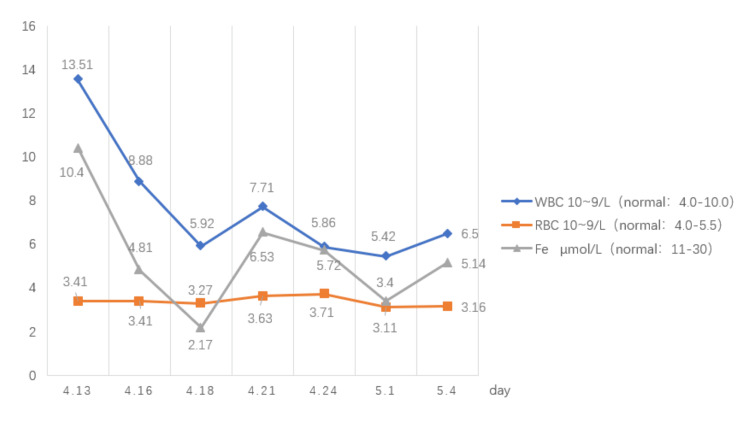
Blood monitoring.

The postoperative pathology showed (bilateral testes and perineum-infected tissue) fibrous tissue hyperplasia, with a large amount of acute and chronic inflammatory cell infiltration, vascular hyperplasia, congestion, focal necrosis, and small abscess formation (Figure 7). Calcitoninogen status and various blood indices of the patients were continuously observed and compared with the preoperative period (Figures 8-9).

**Figure 10:**
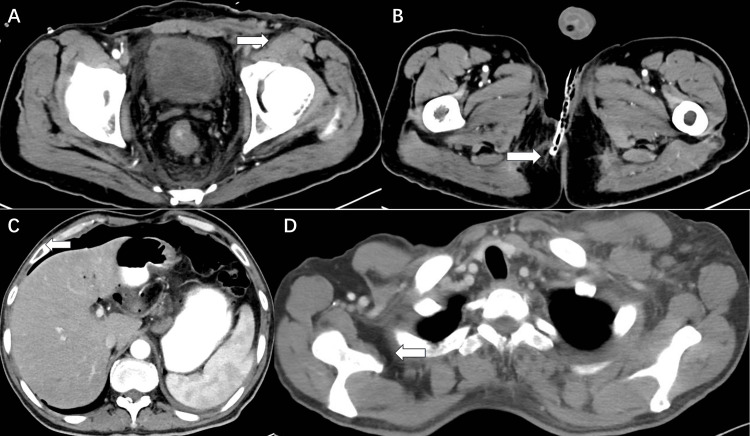
Post-treatment CT images (A-D).

Leukocytes were significantly elevated at the peak of the infection, and red blood cells and serum iron levels were reduced. The patient developed infectious anemia. After discharge, the patient underwent skin grafting at the Qilu Hospital of Shandong Province with good results (Figure 6C). In May 2023, the patient had a follow-up CT（Figures 10A-10D).

## Discussion

An intestinal fistula is an abnormal passage between the intestine and other organs or between the intestine and the abdominal wall. The former is called an internal fistula, and the latter is an external fistula. Intestinal fistulas cause intestinal contents to flow out of the intestinal lumen, resulting in pathophysiological changes, such as infection, fluid loss, malnutrition, and organ dysfunction. The most common causes of intestinal fistula are surgery, trauma, abdominal infection, malignant tumor, radiation injury, chemotherapy, intestinal inflammation, and infectious diseases. The cause of perineal fistula after chemotherapy may be related to localized mucosal necrosis resulting from chemotherapy, thus invasion of the perineum. A rectovaginal fistula (RVF) is very rare in male patients and may manifest as destruction of the scrotum and most perineal soft tissues, that is, Fournier's gangrene. Al-Bahri et al. [5] reported a case of rectal cancer invasion of the perineum secondary to Fournier gangrene in the treatment of an RVF. They also took skin grafts. Fournier's gangrene is a fast progressive necrotizing fasciitis of the perineum and external genitals. It is secondary to polymicrobial infection by aerobic and anaerobic bacteria with synergistic action. The origin of the infection is either cutaneous, urogenital, or colorectal. Jdaini et al. reported a very rare case of cancer of the penis manifested by Fournier's gangrene [6]. It was rare to meet a case of rectal cancer invading so severely in the clinic.

In contrast, it is usually an RVF in females after natural delivery and surgery for rectal cancer [7]. There are more studies on RVF, and most of them have been treated with the same treatment as RVF. There are more studies, most of which require surgical treatment, and various repair styles are fully developed, such as sphincter repair closure [8], flap repair, and biomaterial repair [9]. In the abdominoperineal resection, which also creates a large tissue defect resulting in perineal wound complications, dehiscence, and perineal hernia, reconstructive flaps, such as the vertical rectus abdominis muscle (VRAM) flap, gracilis, anterolateral thigh, and gluteal flaps have been utilized in our institution to address perineal closure[10]. Garcia et al. reported that a patient had a recipient bed (perineum) complication from prior radiation therapy[11].

## Conclusions

In this case, the patient underwent a series of chemotherapies due to rectal cancer and subsequently developed a rectoperineal fistula. By observing the CT and procalcitonin, as well as various blood indices, the patient was found to have a systemic infection, scrotal cellulitis, and testicular necrosis manifesting as Fournier's gangrene. To alleviate the inflammation, he underwent debridement such as bi-orchidectomy. The patient was faced with a huge challenge to the patient's prognosis, both physically and psychologically. Subsequently, it was necessary to combine other disciplines and assist the patient with skin grafting with the help of a plastic surgeon, which was extremely helpful for wound healing. Therefore, the purpose of reporting this case is to raise clinicians' awareness that chemotherapy for rectal cancer can present a wide range of conditions and that clinical medicine needs to continue to broaden its understanding and consider the full range of issues.
